# Volatile organic compound profiling to explore primary graft dysfunction after lung transplantation

**DOI:** 10.1038/s41598-022-05994-2

**Published:** 2022-02-08

**Authors:** Pierre-Hugues Stefanuto, Rosalba Romano, Christiaan A. Rees, Mavra Nasir, Louit Thakuria, Andre Simon, Anna K. Reed, Nandor Marczin, Jane E. Hill

**Affiliations:** 1 Thayer School of Engineering, Dartmouth College, Hanover, NH USA; 2 Organic and Biological Analytical Chemistry Group, Liège University, Liège, Belgium; 3 Department of Surgery and Cancer, Section of Anaesthetics, Imperial College of London, London, UK; 4 Harefield Hospital, Royal Brompton and Harefield NHS Foundation Trust, Harefield, UK; 5 Geisel School of Medicine, Dartmouth College, Hanover, NH USA; 6 Department of Anesthesia and Intensive Care, Semmelweis University, Budapest, Hungary; 7 Department of Chemical and Biological Engineering, University of British Columbia, Vancouver, Canada

**Keywords:** Lipidomics, Metabolomics, Chemical tools, Cheminformatics, Metabolomics, Biomarkers, Medical research

## Abstract

Primary graft dysfunction (PGD) is a major determinant of morbidity and mortality following lung transplantation. Delineating basic mechanisms and molecular signatures of PGD remain a fundamental challenge. This pilot study examines if the pulmonary volatile organic compound (VOC) spectrum relate to PGD and postoperative outcomes. The VOC profiles of 58 bronchoalveolar lavage fluid (BALF) and blind bronchial aspirate samples from 35 transplant patients were extracted using solid-phase-microextraction and analyzed with comprehensive two-dimensional gas chromatography coupled to time-of-flight mass spectrometry. The support vector machine algorithm was used to identify VOCs that could differentiate patients with severe from lower grade PGD. Using 20 statistically significant VOCs from the sample headspace collected immediately after transplantation (< 6 h), severe PGD was differentiable from low PGD with an AUROC of 0.90 and an accuracy of 0.83 on test set samples. The model was somewhat effective for later time points with an AUROC of 0.80. Three major chemical classes in the model were dominated by alkylated hydrocarbons, linear hydrocarbons, and aldehydes in severe PGD samples. These VOCs may have important clinical and mechanistic implications, therefore large-scale study and potential translation to breath analysis is recommended.

## Introduction

Primary graft dysfunction (PGD) is a major complication and leading cause of death following lung transplantation that develops in the early postoperative period (72 h) ^1–4^. PGD reflects the summation of injury inflicted on the donor lung by the transplant process including donor-related factors, preservation and reperfusion injury, intraoperative factors, and consequences of intensive care management. Moreover, in the long term, the PGD score correlates with higher risk of chronic lung allograft dysfunction (CLAD)^3,4^. The incidence of PGD in lung transplant recipients is variable, but approximately 20 percent of patients develop the most severe grade of PGD (grade 3)^5^. Furthermore, PGD is associated with an increased risk of 90-day and 1-year mortality^5^.

Transplant recipients can be risk-stratified based on their clinical diagnosis, the degree of pulmonary hypertension, and body mass index (BMI)^5^. Intraoperative factors such as reperfusion conditions, hyperoxia, and the requirement for conducting the surgery with cardiopulmonary bypass (CPB) have also been identified to significantly impact PGD^5^. There has been some progress in determining the basic mechanisms of PGD and toward identification of genetic or molecular biomarkers capable of predicting and monitoring PGD^6^. Shah and colleagues monitored proteins associated with epithelial injury, coagulation cascade, and cell adhesion. They found that soluble receptors for advanced glycation end products were associated with PGD development^7^. Suberviola and colleagues identified an overexpression of procalcitonin in PGD patients^8^. Upregulated gene expression involving the inflammasome and immune system signaling pathways was reported by Cantu and colleagues^9^ as well as Anraku and colleagues^10^ for patients developing PGD. There is also evidence to suggest that the basic mechanisms of PGD are activated prior to implantation and reperfusion of allografts. In the genetic profiling studies by the Patterson group, various innate immune pathways, as well as those contributing to apoptosis and oxidative stress, were overexpressed in donor lungs at the end of cold ischemia in those patients who subsequently developed PGD later in the postoperative period^11^.

. VOC analysis has already been applied to pathologies similar to PGD and more chronic aspects of lung transplantation. Kuppers and colleagues investigated the breath of patients with chronic allograft failure after transplantation and with chronic lung allograft dysfunction. They detected multiple likely by-products of lipid peroxidation, such as alkanes and aldehydes^12^. Recent research on plasma from lung transplant patients has also shown the importance of putative peroxidation biomarkers. These peroxidation molecules have also been linked to donor smoking history and organ reperfusion conditions, especially during hyperoxia^13–17^. In addition, Boss and colleagues investigated the breath of patients with acute respiratory distress syndromes (ARDS) in adults, a clinical condition with similarities to PGD. They identified three markers: acetaldehyde, octane, and 3-methyl heptane, as potential ARDS markers^18^. These compounds can also consider a consequence of lipid peroxidation.

The primary hypothesis of the study is that volatile molecule profiling of the bronchoalveolar lavage fluid and airway aspirate samples obtained at the end of the transplant surgery will provide novel information on molecular aspects of subsequent PGD, including identifying biomarkers. The first objective was to profile the VOC pattern of airway samples obtained at the end of the transplant operation and to compare molecular patterns between patients who subsequently developed severe PGD (*i.e.,* grade 3) versus those who did not develop or had lower grade PGD (*i.e.,* grade 0–2). Considering that these samples should represent the net effect of donor lung injury and intraoperative stresses on the background of recipient risk factors, the second objective of the study was to delineate the influence of i) donor factors, ii) known intraoperative variables, and iii) recipient risk factors on the observed VOC patterns. We used solid phase micro-extraction coupled to comprehensive two-dimensional gas chromatography coupled to time-of-flight mass spectrometry (SPME-GC × GC-TOFMS) for chemical extraction and analysis, an approach used successfully on similar samples^19–21^.

## Methods

### Ethical approval

Ethical approval was granted by the Riverside NRES committee in London (Research Ethics Committee approval (reference 13/LO/1052)) and the Royal Brompton & Harefield Research office provided NHS Management Permission for research (R&D reference 2013LS001H). All methods were performed in accordance with the relevant guidelines and regulations.

All patients on the active waiting list for bilateral lung transplantation meeting inclusion criteria were provided with a patient information leaflet during hospital attendances, admissions to Harefield Hospital, or they were mailed to their home address. After having had enough time to study the information sheet and discuss the study with research staff, informed consent forms were signed prior to surgery for lung transplantation.

### Clinical diagnosis of PGD

The International Society of Heart and Lung Transplantations (ISHLT) has developed a grading system to classify PGD from 0 to 3^22^. This scoring system is based on: (1) the severity of hypoxemia, and (2) the detection of lung infiltrate on chest radiograph (with the exclusion of any other cause of hypoxemia). All blood gases data and the corresponding FiO_2_ from the arrival in ICU to 72 h later were recorded and the lowest pO_2_/FiO_2_ ratio was considered for the diagnosis and grading of PGD. Chest X-rays performed within 72 h were reviewed by an expert radiologist. The patient population was split between grade 3 PGD (PGD3) and the lower grade or absence of PGD (*i.e.,* PGD grade 0, 1, or 2; PGD0-2). This stratification scheme has been used in previous studies to underline the clinical relevance of grade 3 PGD for predicting mortality^5,23–25^.

### Sample collection

**Figure 1:**
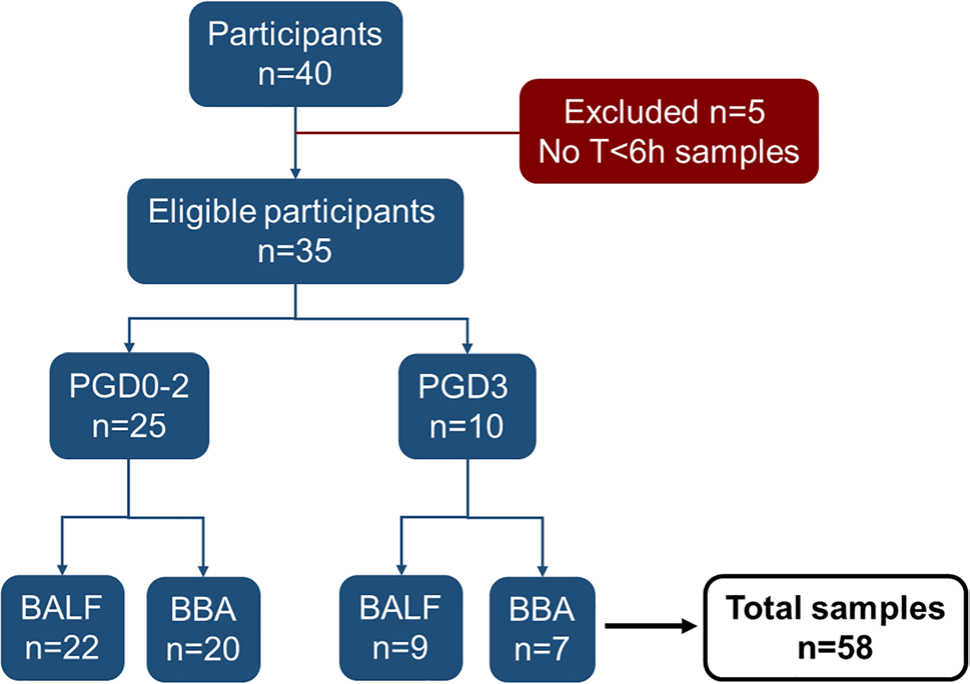
Flow chart of patient population according to Standards for Reporting of Diagnostic Accuracy (STARD) guidelines^26^.

Fiberoptic bronchoscopy is performed immediately after surgery to inspect the bronchial anastomoses immediately before the patient leaves the operating theatre. Small volume bronchial washings (BALF) were performed by the transplant surgeon by instilling 20 mL of normal saline via the bronchoscope and aspirated into suction traps. Blind bronchial aspirates (BBA) were taken at the same time. The patient and sample population is described according to Standards for Reporting of Diagnostic Accuracy (STARD) guidelines in Fig. 1^26^. In order to evaluate the potential of PGD prediction, the initial model was built on samples taken at the end of the surgery after reperfusion of lung allografts or shortly after arrival to the intensive care unit (T < 6 h).

### Analytical conditions

Volatile organic compounds released from the headspace of lung fluid samples were concentrated onto solid phase micro-extraction and two-dimensional gas chromatography coupled to a time-of-flight mass spectrometer (HS-SPME-GC × GC-TOFMS)^19,27–29^. Analytical parameters are provided in supplementary materials. The details of the pre-processing and compound identification are also provided in supplementary materials.

### Processing: model building and feature selection

**Figure 2:**
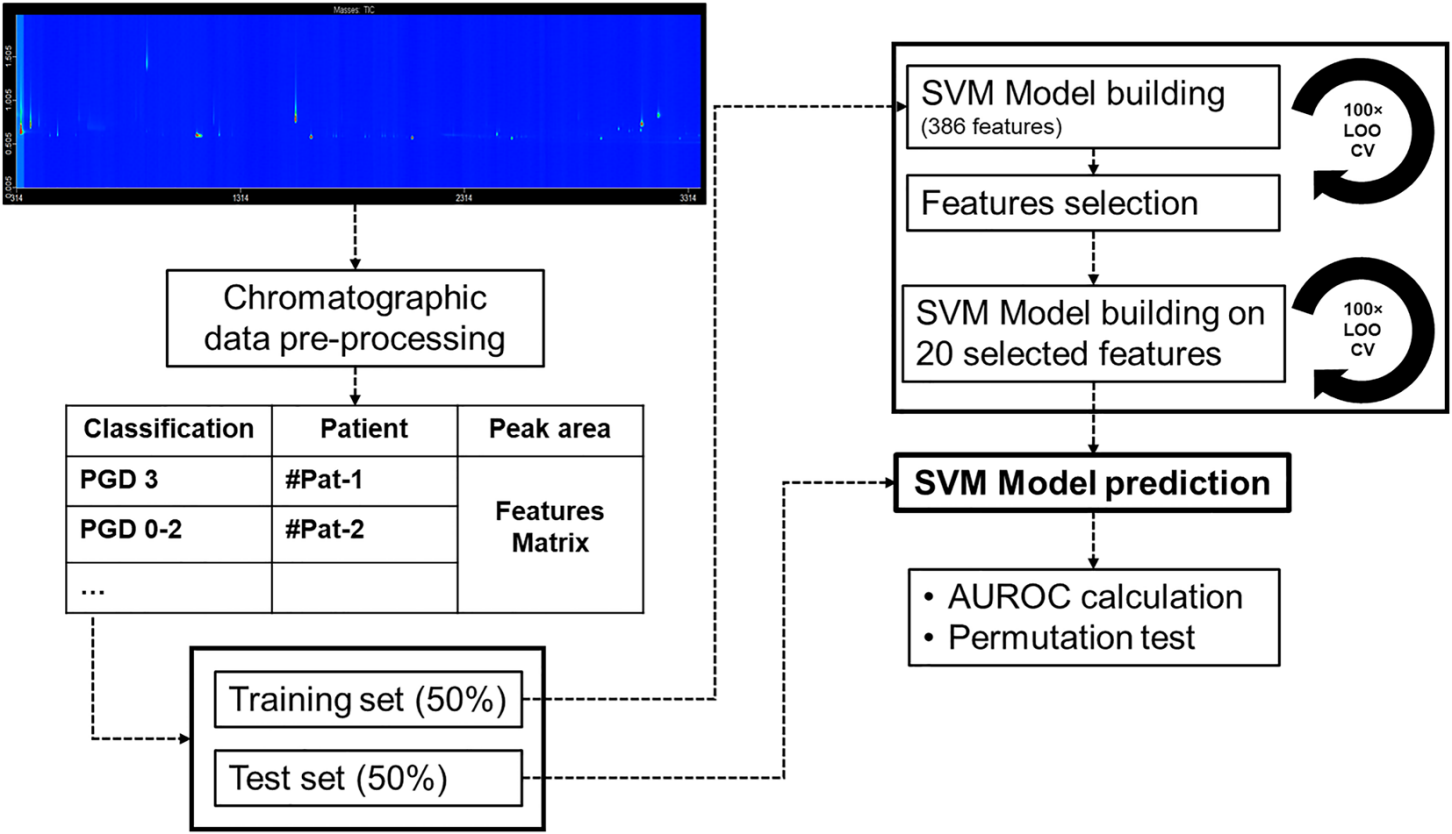
Feature selection, model building, and prediction. LOOCV: leave-one-out cross validation; SVM: support vector machine; AUROC: area under receiver operating characteristic.

Due to the small population of this study, different cross-validation approaches were tested in order to build a robust classification model. Each method was tested for over-fitting using random label and random feature selection. Both the no cross validation and simple leave-one out cross validation approaches overfit the data, providing a non-significant no information rate (*p* > 0.05). Therefore, to limit overfitting, the data were split between a training and a test set with a 1:1 ratio. For the data splitting, all the samples from a single patient were kept in the same group to avoid overfitting. The model building and the feature selection were performed using support vector machine (SVM) with a linear kernel on the training set (*i.e.,* eight PGD3 subjects and twenty PGD0-2 subjects, Fig. 2). SVM was selected for its ability to create a classification model from a high dimensional data set, (*i.e.,* with limited number of observation and large number of variables)^30,31^. The model used a linear kernel in order to avoid data overfitting generally observed with polynomial approaches. With a linear approach, the vector separating the data is a straight line. The feature importance was scaled to 100 (with 100 representing the most discriminatory feature). Features with scaled importance above 50 were selected. Moreover, to be kept in the model a feature has to be found in a least 25% of the samples from one class (*i.e.,* PGD3 or PGD0-2). Based on these criteria, 20 features were selected from the initial 386, and a SVM model was built from these to discriminate between PGD3 and PGD0-2. The model was subsequently evaluated on the test set, using area under the receiver operating curve (AUROC). To evaluate potential over-fitting and batch effects, random label and random feature selection tests were performed and conducted to random classification (Figure SI-1). The complete data processing flow can be found in Fig. 2.

All statistical analyses were performed in R version 3.6.1 (The R Foundation for Statistical Computing)^32^. More information about the data processing and the R packages used is provided in supplementary materials.

### Influence of clinical factors on VOC profiles

The impact of various donor, recipient, and procedural risk factors on the volatile profile composition were evaluated. A multivariate analysis of variance (MANOVA) using the categorical clinical factors and the VOC pattern from samples was conducted. MANOVA is a way to test the hypothesis that one or more independent variables, or factors, have an effect on a set of two or more dependent variables.

## Results

### Study population

The population characteristics are provided in Table 1. Out of the 35 lung transplant recipients, 10 (28%) developed grade 3 PGD. While donor and recipient and most surgical factors were comparable, the use of Organ Care System (OCS) was higher in the PGD3 group (*p* < 0.05). Patients with PGD3 required a longer duration of mechanical ventilation (*p* < 0.05) and generally stayed longer in the Intensive Care Unit and in the hospital (Table 1).

**Table 1:** Description of the study population.

	Study group		*p*-value
	PGD 0–2 (n = 25)	PGD 3 (n = 10)	PGD 3 versus PGD 0–2
**Donor factors**			
Donor age	39 (14)	48 (16)	0.1
Donor weight	73 (15)	76 (27)	0.9
Donor height (cm)	169 (9)	172 (11)	0.4
Donor BMI	26 (5)	25 (7)	0.5
Cause of death: Trauma; n (%)	2 (8%)	3 (30%)	0.09
Male Donor gender; n (%)	13 (52%)	5 (50%)	0.9
DCD transplant; n (%)	7 (28%)	2 (20%)	0.6
**Recipient factors**			
Recipient age	48 (16)	54 (10)	0.4
Male Recipient; n (%)	10 (40%)	6 (60%)	0.3
Underlying disease			
Cystic fibrosis	7 (28%)	2 (20%)	0.6
COPD-Emphysema	14 (56%)	6 (60%)	0.8
Others	4 (16%)	2 (20%)	0.8
High-risk for PGD 3^a^	11 (44%)	8 (80%)	0.07
**Surgical factors**			
Surgical approach: MILT n (%)^b^	8 (32%)	5 (50%)	0.3
CPB; (n (%))	8 (32%)	5 (50%)	0.3
OCS (n (%))	1 (4%)	3 (30%)	**0.03**
**Outcomes**			
1 year Mortality; n (%)	1 (4%)	2 (20%)	–
Ventilation (h)	143 (316)	443 (414)	**0.003**
ICU LOS after Tx (days)	9 (13)	21 (18)	**0.06**
Hospital LOS after Tx (days)	38 (28)	48 (33)	0.3
**Lung function at 3 months**	(n = 23)	(n = 9)	
FVC (% predicted)	71.0 (21.2)	68.4 (31.4)	0.8
FEV1 (% predicted)	75.0 (24.8)	73.4 (30.8)	1.0
MEFR (% predicted)	87.3 (27.9)	83.0 (27.9)	0.6
75%FVC (% predicted)	84.7 (31.4)	76.9 (21.6)	0.7
50%FVC (% predicted)	89.1 (47.0)	82.3 (27.1)	0.8
25%FVC (% predicted)	115.1 (100.7)	89.2 (14.9)	0.8
MIFR (% predicted)	89.3 (46.4)	93.4 (38.3)	0.7
FEV1/FVC ratio	88.7 (12.3)	90.4 (7.2)	0.9

The Wilcoxon test was applied for continuous variables and the Chi-squared for the categorical variables. For continuous variables, the values in the table represent the mean for each group, with the standard deviation in brackets. The non-aggregated data are available in Supplementary Materials.

*BMI* body mass index, *DCD* donor after circulatory death, *DBD* donor after brain death, *PGD* primary graft dysfunction, *CPB* cardiopulmonary bypass, *OCS* organ care system, *LOS* length of stay, *FVC* forced vital capacity, *FEV1* forced expiratory volume in the 1st second, *MEFR* maximal expiratory flow rate, *MIFR* maximal inspiratory flow rate.

^a^The high-risks for PGD 3 are based on Shah et al.^33^.

^b^MILT: Minimally Invasive Lung Transplant (performed instead of the more traditional Clamshell approach).

Significant values are in [bold].

### Volatile organic compounds panel comparison between BALF and BBA

This study evaluated two lung fluids, BALF and BBA. The correlation between the two different matrices from the same patient at the same sampling point was evaluated using a Pearson’s correlation calculation. The average Pearson’s correlation coefficient between a BALF-BBA pair from a single individual (0.65) was higher than within a sample type across all individuals (BALF (0.53) and BBA (0.51)). In addition, a Spearman’s correlation for the same combination was also calculated. The average correlations obtained were almost identical to the ones from Pearson’s correlation: BALF-BBA (0.65); BALF (0.55); and BBA (0.53) with a standard deviation of less than 0.09. This indicates that pair sample for the same individual contains a similar VOC profile, and that the ranking of the VOC in absolute intensity (Pearson) and in rank order (Spearman) are also similar. Based on this observation, two matrices collected from a single individual were processed together, as the within-individual variation was substantially less than the between-individual variation.

### Model building and VOC feature selection

**Figure 3:**
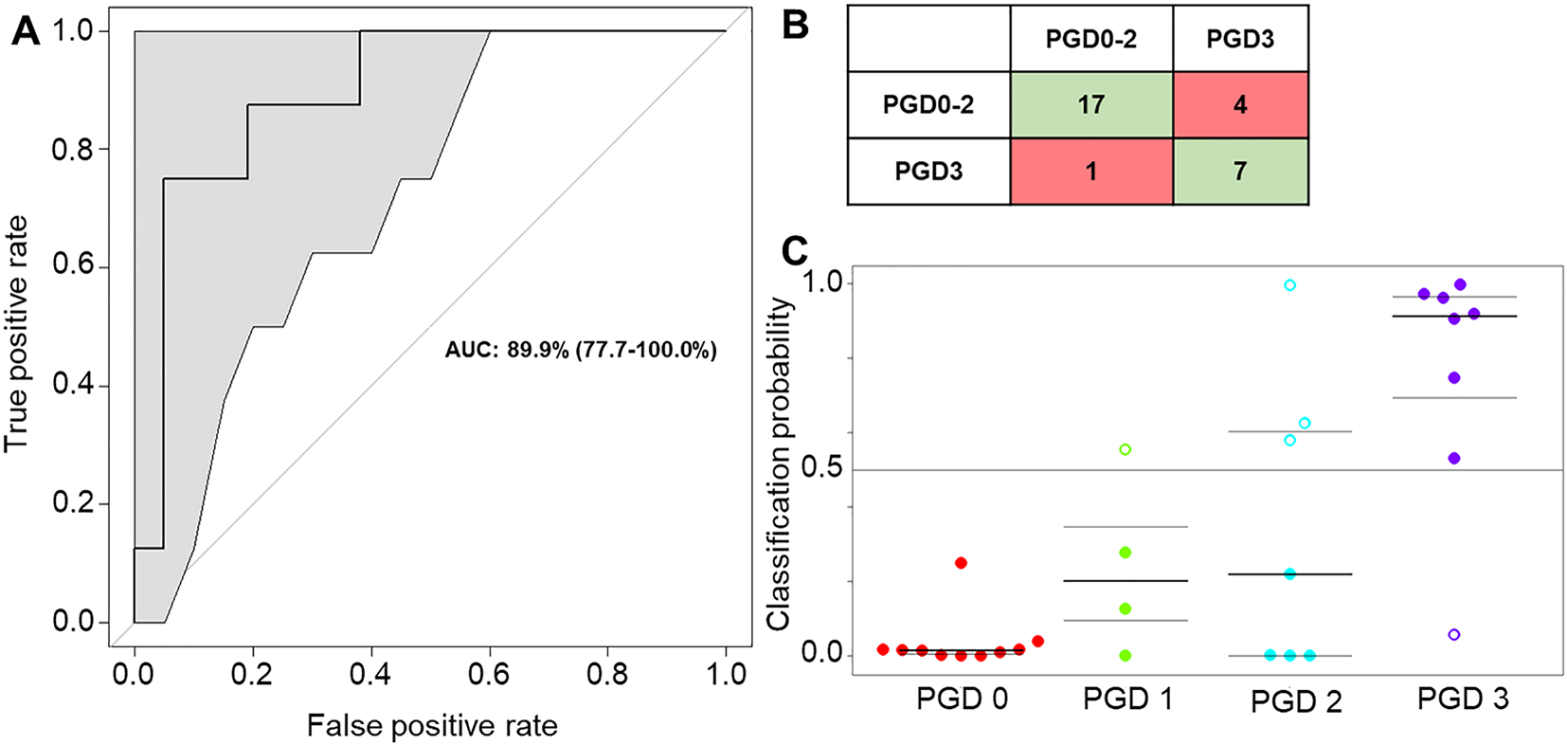
(**A**) Receiver operating characteristic (ROC) curve with 95% confidence interval (grey shape) on the test set. (**B**) Confusion matrix for the test set on the selected features model. The green boxes correspond to correct classification, the red ones to misclassification. (**C**) Classification probability according to the clinical PGD. The filled dots correspond to correct classification, the empty ones to misclassification. Figures of merit are provided in Table SI-2.

To generate the model at time just after transplant (< 6 h), the number of molecules from sample headspace was reduced from 386 features (Fig. 2 and Table SI-1) to 20 selected features using an SVM model targeting discrimination between grade 0–2 and grade 3 PGD. The test set AUROC from the 20 selected features reached 0.90 (95% confidence interval: 0.77–1.00) and an accuracy of 0.83 (95% confidence interval: 0.64–0.94) (Fig. 3A). The classification accuracy is reflected in the confusion matrix (Fig. 3B); there, four PGD0-2 patient samples misclassified as PGD3, specifically, three grade 2 and one grade 1 (Fig. 3C). The SVM model had a sensitivity of 0.63, specificity of 0.94, positive predictive value of 0.87, and negative predictive value of 0.80. These indicators are summarized in Table SI-2.

Permutation testing of the model demonstrated the non-random efficiency of the selected features panel. In a first approach, the model creation-prediction step was repeated on 20 randomly selected features, generating an AUROC of 0.49 (Figure SI-1). In a second approach, random labels were used in the test set using the 20 discriminatory features. The resulting AUROC was also 0.49 (Figure SI-1). In both cases, resulting AUROCs indicated classification of patients at random, confirming the lack of bias in data processing.

The model performance was also evaluated at later time points (6–72 h) post-transplant, specifically seven grade 3 PGD and seven grade 0–2 PGD patient samples. The resulting AUROC was 0.80. Performance indicators for this model can be found in Table SI-3.

### Identification of molecules in model

**Figure 4:**
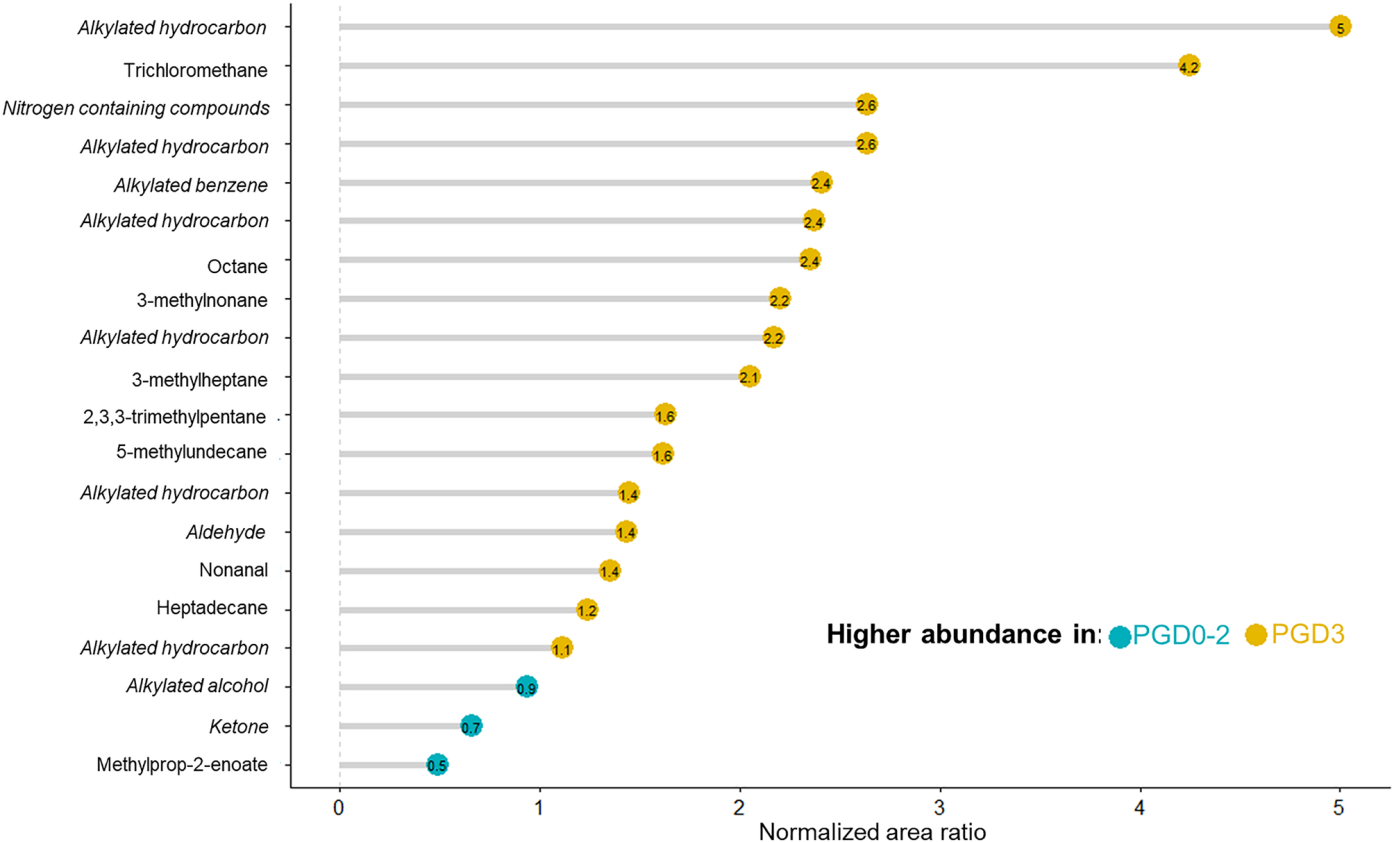
Ratio of normalized area of the 20 selected features.

From 20 features, nine met the specified identification criteria and were putatively named (see supplementary materials and Table SI-1)^34^. The remaining 11 features were given a chemical family name, only (*italicized* in Fig. 4)^35^. The normalized area ratio shows that the majority of the signals are more abundant in the grade 3 PGD group compared to the lower grade samples. Only three signals were more abundant in the grade 0–2 PGD group.

#### Influence of clinical risk factors on VOC patterns

There was no statistical relationship between VOC pattern and type of donation (brain or cardiac determination of death) or cause of donor death between head trauma and other causes. However, the donor BMI (classified as low, normal, or high, according to recommended cutoffs) correlates with the volatile profile (*p* < 0.05), even if the continuous values do not show significant difference (*p* = 0.51). The use of the Organ Care System correlated with the selected features used to build the PGD classification model (*p* < 0.05). The invasiveness of the surgical technique (comparing the minimally-invasive transplant performed through thoracotomies rather than the more invasive clamshell approach) and the use of cardiopulmonary bypass had no major influence on VOC signatures (*p* > 0.05).

The total ischemia time was similar (*p* = 0.54) between the grade 3 PGD and grade 0–2 PGD populations by the Wilcoxon test. MANOVA showed no statistically significant relationship between the volatile compounds and the stratified ischemia times (*p* > 0.05; ischemia times were stratified into short (< 6 h), medium (6–10 h) and long (> 10 h)). To investigate PGD recipient risk and the 20 molecule VOC pattern, patients were classified into low or high recipient risk^33^. The low-risk recipient group was composed of 16 subjects who were characterized by normal BMI (18.5–25 kg/m^2^), the diagnosis of COPD/CF, and absent or mild pulmonary hypertension, leaving 19 patients as high risk recipients. No clustering was observed according to recipient risk factor and no significant difference is observed through the MANOVA analysis. The potential relationship between the VOCs and pulmonary function beyond PGD was investigated by evaluating the correlation between the selected features and the lung function after three months. No significant relationship was identified with either the intraoperative samples or later postoperative BALF or BBA VOC samples (*p* > 0.05). This non correlation is perhaps linked to the small and unbalanced population for some of the risk factors. The next phase of the study, on a larger population, should provide more insights on potential relationships between VOC and CLAD development.

## Discussion

This pilot study extends current efforts toward uncovering basic mechanisms and biological markers of lung injury following lung transplantation. It suggests that the paradigm that clinical manifestations of severe PGD are preceded by biochemical alterations that can be characterized by a unique pattern of volatile molecular signatures in the headspace of alveolar and bronchial fluids. In order to justify this conclusion, we shall discuss the following aspects: the patient population; timing and nature of biological samples; the analytical, statistical and chemical aspects of the molecular profiles; the clinical correlates with the volatile profiles; and limitations of the study in each of these domains.

The population of this pilot study approximates the wider lung transplant demographic both at our institution and globally. The recipient diagnoses have a spectrum of clinical risk profiles for the development of PGD and the previously reported prevalence of approximately 25–30% for grade 3 PGD^5^. The development of PGD was associated with poorer clinical outcomes as represented by longer ventilation times and total length of stay in the ICU (Table 1). ICU duration is highly correlated (0.96) with ventilation time. The study was not designed to elucidate the clinical determinants of PGD, however, no obvious donor or recipient characteristic was associated with the development of PGD. Intraoperative factors such as cardiopulmonary bypass and invasiveness of surgery were investigated, as these aspects have been suggested as potential risk factors^5,36^. Though not statistically significant, there was a tendency for higher use of cardiopulmonary bypass (CPB) in the severe PGD group, which is in-line with previous literature data^5^ and our own wider experience^36,37^. In contrast, the association with Organ Care System use (Table 1) is surprising as the more global experience with OCS has been reduction in severe PGD during the first 72 h after lung transplantation by the use of OCS as reported by the recent INSPIRE trial^38,39^. However, the multicenter INSPIRE trial focused on standard criteria donors, whereas our initial OCS experience mainly included marginal or extended criteria donor lungs with potentially worse outcomes and an initial learning curve with the technology^40,41^.

This study utilized two types of lung fluid samples, BALF and BBA. An important part of the study was dedicated to the comparison of the volatile profile of these two matrices. A correlation (Pearson approach) was observed between the VOC composition for the two matrices within a patient at the same sampling time. This observation allowed us to chemometrically process BALF and BBA sample data together and utilize the VOC composition of both matrices for the model development. Such similarities of the VOC pattern raise the intriguing question as to whether the VOCs pattern of exhaled breath would also be similar to airway fluid samples.

The most significant finding of the current study is the potential association of severe PGD shortly after lung implantation and reperfusion with a unique VOC profile. And, that this profile was present through the early postoperative period. The model identified patients who subsequently developed grade 3 PGD with an AUROC of 0.90 and a positive predictive value of 0.88, which compare favorably to previous biomarkers in plasma. This volatile molecule approach represents a novel molecular strategy for the detection and monitoring of allograft injury. Although these results require further validation, identification of PGD patients immediately after transplantation, may allow for the implementation of lung protective strategies and earlier treatment of PGD^25^. Nine different chemical families were identified from the selected 20 volatile signature: ten alkylated hydrocarbons, two linear hydrocarbons, and two aldehydes (see Table SI-4). These molecules were more abundant in severe PGD patients. It is interesting to link these observations with the recent findings of Kuppers and colleagues, who investigated chronic allograft failure after transplantation and found similar chemical families in the breath of patients with chronic lung allograft dysfunction^12^. The production of linear hydrocarbons and the aldehydes are considered likely by-products of lipid peroxidation. For example, plasma lipid peroxidation biomarkers are expected to be produced during in lung transplantation and have also been linked to donor smoking history and reperfusion conditions, especially hyperoxia^13–17^. Alkylated hydrocarbons have been repeatedly detected in breath samples of diseased subjects^18,42^, although there are no specific metabolic pathways that can precisely account for the production of these compounds in these and other contexts without conformational testing^18^. Here, the origin of the volatile molecules is unknown (*e.g.,* potential metabolic pathways, environmental factors, endogenous origins). It is crucial to ultimately identify the origin of these highly abundant compounds in order to generate insights into pathogenesis processes. Taken together, these studies further support the potential place of VOC analysis at various injury states after lung transplantation. The identification of octane and 3-methylheptane as significant discriminatory elements is interesting. These two compounds were previously identified as potential breath biomarkers of acute respiratory distress syndromes (ARDS) in adults^18^. The independent identification of these compounds in patients with severe PGD is particularly encouraging based on the similarities between these two clinical conditions. Furthermore, as octane has been directly linked to lipid peroxidation^43^, this may represent a further argument for the role of oxidative stress in lung injury following lung transplantation.

The second objective of the study was to analyze the potential influence of various donor, recipient and procedural factors of lung transplantation on the VOC composition and the 20 identified VOC features. Among the donor characteristics, donor BMI correlates with the volatile profile. Currently, there is a strong debate regarding the negative and positive influence of obesity on the development of acute lung injury, with both injurious and protective mechanisms proposed^44^. These data indicate that the perioperative VOC PGD pattern might be linked to preexisting donor. One can also hypothesize that the mode of donor death such as trauma, brain hemorrhage or cardiac death may affect the lung inflammatory milieu. However, the clinical evidence suggests that transplantation from these sources have similar outcomes to control donors. The VOC data may help to provide a molecular explanation for these clinical paradigms. A relationship could also be expected between emerging clinical risks of PGD and VOC patterns (Table 1). Surprisingly, higher risk recipients, or the use of CPB had no statistically significant influence on the 20 molecule VOC pattern. There may be several explanations for these findings. As the recipient risk is computed with different diagnostic and physiological variables, these may become significant with a much larger patient population. While the role of CPB in the development of PGD has emerged, there are major differences between institutions regarding the clinical use and conduct of CPB. This study population represents a transitional phase of lung transplantation between routine elective CPB and off-pump transplantation with short and uncomplicated CPB runs, where the influence of CPB remains more controversial^45^. In the current study, the minimally invasive surgical approach (MILT) did not seem to influence the VOC pattern when compared to the invasive clamshell incision. This seems to resonate with previous findings, whereby MILT had little influence on clinical inflammatory parameters, despite having a beneficial postoperative impact^36^. While ischemia time itself showed no correlation with the features through MANOVA testing, there was a relationship with the use of OCS. This preliminary finding is interesting, but this could be related to the inclusion of marginal donor lungs as a major indication for the OCS evaluation. This would correlate with earlier clinical reports with a tendency of higher PGD and ECMO requirement associated with early OCS experience^41^. Alternatively, the differential VOC pattern could be the result of a wider inflammatory and metabolic milieu associated with current practice of ex vivo perfusion, as recently demonstrated during the DEVELOP-UK trial^46^. Although these preliminary observations regarding the clinical correlates and VOC profiles are of interest, the potential influence of confounding factors such as pathogens and infections, brain death, inflammation cannot be excluded. Future, larger studies, with defined populations, longitudinal sampling, variable transplant approaches, etc. will evaluate the extent to which these proposed biomarkers are useful to the lung transplant field for transplant rejection as well as a greater understanding of disease processes.

The main limitation of this study is the small study population (35 patients). We believe that we have conducted an essential exploratory study with a patient cohort appropriate for pilot biochemical and hypothesis generating studies, and now drives the development of a larger study. Another limitation is the absence of exhaled breath analysis. While we have pioneered breath nitric oxide studies in lung transplant recipients in the perioperative setting and in infectious etiologies^29,47–49^, breath analysis of VOCs in the operating environment remains challenging. Nevertheless, there has been progress in this field, for instance the measurement of ethylene (a lipid peroxidation product), in cardiac surgical and critically ill patients^50^ and by the application of direct MS to this environment^51^. The data from this study suggests that breath should also be considered in future studies. Furthermore, the full identification and quantification of the selected features will require the utilization of additional analytical tools, such as high-resolution mass spectrometry. Despite these limitations, these results demonstrate an important potential for volatile molecule profiling to assess and monitor the development of PGD, one of the most important clinical limitation of current lung transplantation^52^.

In conclusion, the analysis of volatile molecules from BALF and BBA samples and their association with PGD at the time of lung transplantation offers valuable mechanistic insights of ongoing metabolism in the lungs. The selected features highlight the potential importance of lipid peroxidation. The selected features open the route to further investigations on the specific metabolism pathways active in patients with severe PGD. These data suggest that lung fluid analysis may have high clinical applicability and monitoring potential and it should be investigated in a larger scale study. The potential translation to breath volatile analysis should be investigated towards implementation of non-invasive molecular assessment.

## Supplementary Information

Supplementary Information 1.

Supplementary Information 2.

## Data Availability

The datasets generated and/or analyzed during the current study are available from the corresponding author on reasonable request.

## References

[1] Christie JD, Carby M, Bag R, Corris P, Hertz M, Weill D (2005). Report of the ISHLT working group on primary lung graft dysfunction part II: Definition. A consensus statement of the international society for heart and lung transplantation. J. Heart Lung Transpl..

[2] Christie JD, Edwards LB, Kucheryavaya AY (2010). The registry of the international society for heart and lung transplantation: Twenty-seventh official adult lung and heart-lung transplant report2010. J. Heart Lung Transpl..

[3] Christie JD, Sager JS, Kimmel SE (2005). Impact of primary graft failure on outcomes following lung transplantation. Chest.

[4] Daud SA, Yusen RD, Meyers BF (2007). Impact of immediate primary lung allograft dysfunction on bronchiolitis obliterans syndrome. Am. J. Respir. Crit. Care Med..

[5] Diamond JM, Lee JC, Kawut SM (2013). Clinical risk factors for primary graft dysfunction after lung transplantation. Am. J. Respir Crit. Care Med..

[6] Lee GM (2009). Early post-mortem changes and stages of decomposition in exposed cadavers. Exp. Appl. Acarol..

[7] Christie JD, Shah CV, Kawut SM (2009). Plasma levels of receptor for advanced glycation end products, blood transfusion, and risk of primary graft dysfunction. Am. J. Respir. Crit. Care Med..

[8] Suberviola B, Rellan L, Riera J (2017). Role of biomarkers in early infectious complications after lung transplantation. PLoS ONE.

[9] Cantu E, Lederer DJ, Meyer K (2013). Gene set enrichment analysis identifies key innate immune pathways in primary graft dysfunction after lung transplantation. Am. J. Transpl..

[10] Anraku M, Cameron MJ, Waddell TK (2008). Impact of human donor lung gene expression profiles on survival after lung transplantation: A case-control study. Am. J. Transpl..

[11] Ray M, Dharmarajan S, Freudenberg J, Zhang W, Patterson GA (2007). Expression profiling of human donor lungs to understand primary graft dysfunction after lung transplantation. Am. J. Transpl..

[12] Kuppers L, Holz O, Schuchardt S (2018). Breath volatile organic compounds of lung transplant recipients with and without chronic lung allograft dysfunction. J. Breath Res..

[13] Sato K, Kadiiska MB, Ghio AJ (2002). In vivo lipid-derived free radical formation by NADPH oxidase in acute lung injury induced by lipopolysaccharide: A model for ARDS. FASEB J..

[14] Frank Kneepkens CM, Lepage G, Roy CC (1994). The potential of the hydrocarbon breath test as a measure of lipid peroxidation. Free Radic. Biol. Med..

[15] Diamond JM, Porteous MK, Jackson Roberts L (2016). The relationship between plasma lipid peroxidation products and primary graft dysfunction after lung transplantation is modified by donor smoking and reperfusion hyperoxia. J. Heart Lung Transpl..

[16] Grob NM, Aytekin M, Dweik RA (2008). Biomarkers in exhaled breath condensate: A review of collection, processing and analysis. J. Breath Res..

[17] Cikach FS, Dweik RA (2012). Cardiovascular biomarkers in exhaled breath. Prog. Cardiovasc. Dis..

[18] Bos LDJ, Weda H, Wang Y (2014). Exhaled breath metabolomics as a noninvasive diagnostic tool for acute respiratory distress syndrome. Eur.. Respir J..

[19] Nasir M, Bean HD, Smolinska A, Rees CA, Zemanick ET, Hill JE (2018). Volatile molecules from bronchoalveolar lavage fluid can “rule-in” Pseudomonas aeruginosa and “rule-out” Staphylococcus aureus infections in cystic fibrosis patients. Sci. Rep..

[20] Zanella D, Henket M, Schleich F (2020). Comparison of the effect of chemically and biologically induced inflammation on the volatile metabolite production of lung epithelial cells by GC×GC-TOFMS. Analyst..

[21] Beauchamp, J., Davis, C. & Pleil, J. *Breathborne Biomarkers and the Human Volatilome*. 2nd Edition. (Beauchamp, J., Davis, C., Pleil, J., eds.). Elsevier B.V. (2020).

[22] Snell GI, Yusen RD, Weill D (2017). Report of the ISHLT working group on primary lung graft dysfunction, part I: Definition and grading—A 2016 Consensus Group statement of the International Society for Heart and Lung Transplantation. J. Heart Lung Transpl..

[23] Prekker ME, Nath DS, Walker AR (2006). Validation of the proposed international society for heart and lung transplantation grading system for primary graft dysfunction after lung transplantation. J. Heart Lung Transpl..

[24] Shah RJ, Bellamy SL, Localio AR (2012). A panel of lung injury biomarkers enhances the definition of primary graft dysfunction (PGD) after lung transplantation. J. Heart Lung Transpl..

[25] Pottecher J, Roche A-C, Dégot T (2017). Increased extravascular lung water and plasma biomarkers of acute lung injury precede oxygenation impairment in primary graft dysfunction after lung transplantation. Transplantation.

[26] Bossuyt PM, Reitsma JB, Bruns DE (2003). Towards complete and, accurate reporting of studies of diagnostic accuracy: The STARD initiative. Br. Med. J..

[27] Rees CA, Stefanuto P-H, Beattie SR, Bultaman KM, Cramer RA, Hill JE (2017). Snif fi ng out the hypoxia volatile metabolic signature of Aspergillus fumigatus. J. Breath..

[28] Rees CA, Nordick KV, Franchina FA, Lewis AE, Hirsch EB, Hill JE (2017). Volatile metabolic diversity of Klebsiella pneumoniae in nutrient- replete conditions. Metabolomics.

[29] Mellors TR, Nasir M, Franchina FA (2018). Identification of Mycobacterium tuberculosis using volatile biomarkers in culture and exhaled breath. J. Breath Res..

[30] Guyon I, Weston J, Barnhill S, Vapnik V (2002). Gene selection for cancer classification using support vector machines. Mach. Learn..

[31] Robroeks CM, Van Berkel JJ, Jöbsis Q (2013). Exhaled volatile organic compounds predict exacerbations of childhood asthma in a 1-year prospective study. Eur. Respir. J..

[32] Team RC. R: A language and environment for statistical computing. R Foundation for Statistical Computing (2019).

[33] Shah RJ, Diamond JM, Cantu E (2015). Objective estimates improve risk stratification for primary graft dysfunction after lung transplantation. Am. J. Transpl..

[34] Sumner LW, Amberg A, Barrett D (2007). Proposed minimum reporting standards for chemical analysis: Chemical Analysis Working Group (CAWG) Metabolomics Standards Initiative (MSI). Metabolomics.

[35] Dimandja JMD, Clouden GC, Colón I, Focant JF, Cabey WV, Parry RC (2003). Standardized test mixture for the characterization of comprehensive two-dimensional gas chromatography columns: The Phillips mix. J. Chromatogr..

[36] Marczin N, Popov AF, Zych B (2016). Outcomes of minimally invasive lung transplantation in a single centre: The routine approach for the future or do we still need clamshell incision?. Interact. Cardiovasc. Thorac. Surg..

[37] Sabashnikov A, Weymann A, Mohite PN (2014). Risk factors predictive of one-year mortality after lung transplantation. Eur. J. Cardio-thoracic. Surg..

[38] Loor G, Warnecke G, Smith M (2016). The OCS Lung EXPAND international trial interim results. J. Heart Lung Transpl..

[39] Warnecke G, Van Raemdonck D, Smith MA (2018). Normothermic ex-vivo preservation with the portable Organ Care System Lung device for bilateral lung transplantation (INSPIRE): A randomised, open-label, non-inferiority, phase 3 study. Lancet Respir. Med..

[40] Mohite PN, Maunz O, Popov AF, Zych B, Patil NP, Simon AR (2015). Utilization of the organ care system as ex-vivo lung perfusion after cold storage transportation. Perfus (United Kingdom)..

[41] Zeriouh M, Sabashnikov A, Mohite PN (2016). Utilization of the organ care system for bilateral lung transplantation: Preliminary results. Interact. Cardiovasc. Thorac. Surg..

[42] Filipiak W, Mochalski P, Filipiak A (2016). A compendium of volatile organic compounds (VOCs) released by human cell lines. Curr. Med. Chem..

[43] Bos LDJ (2018). Diagnosis of acute respiratory distress syndrome by exhaled breath analysis. Ann. Transl. Med..

[44] Zhi G, Xin W, Ying W, Guohong X, Shuying L (2016). “ Obesity Paradox ” in acute respiratory distress syndrome : Asystematic review and meta-analysis. PLoS ONE.

[45] Nandor M, Royston D, Yacoub M (2000). Pro: Lung transplantation should be routinely performed with cardiopulmonary bypass. J. Cardiothorac. Vasc. Anesth..

[46] Fisher A, Andreasson A, Chrysos A (2016). An observational study of Donor Ex Vivo Lung Perfusion in UK lung transplantation: DEVELOP-UK. Health Technol. Assess..

[47] Beccaria M, Mellors TR, Petion JS (2018). Preliminary investigation of human exhaled breath for tuberculosis diagnosis by multidimensional gas chromatography: Time of flight mass spectrometry and machine learning. J. Chromatogr. B Anal. Technol. Biomed. Life Sci..

[48] Beccaria M, Bobak C, Maitshotlo B (2018). Exhaled human breath analysis in active pulmonary tuberculosis diagnostics by comprehensive gas chromatography-mass spectrometry and chemometric techniques. J. Breath Res..

[49] Marczin N, Riedel B, Gal J, Polak J, Yacoub M (1997). Exhaled nitric oxide during lung transplantation. Lancet.

[50] Romano R, Cristescu SM, Risby TH, Marczin N (2018). Lipid peroxidation in cardiac surgery: Towards consensus on biomonitoring, diagnostic tools and therapeutic implementation. J. Breath Res..

[51] Boshier PR, Cushnir JR, Mistry V (2011). On-line, real time monitoring of exhaled trace gases by SIFT-MS in the perioperative setting: A feasibility study. Analyst..

[52] Diamond JM, Cantu E, Porteous MK (2017). Peripheral blood gene expression changes associated with primary graft dysfunction after lung transplantation. Am. J. Transpl..

