# Supplementary material for: Volatile organic compound profiling to explore primary graft dysfunction after lung transplantation
Source: Sci Rep. 2022 Feb 8;12:2053. doi: 10.1038/s41598-022-05994-2 (PMC8827074; doi:10.1038/s41598-022-05994-2)
Supplement: Supplementary file 1 — Supplementary Information 1. [file 41598_2022_5994_MOESM1_ESM.docx]

**Supplementary materials**

VOLATILE ORGANIC COMPOUND PROFILING TO EXPLORE PRIMARY GRAFT DYSFUNCTION AFTER LUNG TRANSPLANTATION

Pierre-Hugues Stefanuto^1,2#^, Rosalba Romano^3,4#^, Christiaan A. Rees^5^, Mavra Nasir^5^, Louit Thakuria^4^, Andre Simon^4^, Anna K. Reed^4^, Nandor Marczin^3,4,6^ , Jane E. Hill^1,5*^

^1^ Thayer School of Engineering, Dartmouth College, Hanover NH, USA

^2^ Organic and Biological Analytical Chemistry Group, Liège University, Belgium

^3^ Department of Surgery & Cancer, Section of Anaesthetics, Imperial College of London, UK

^4^ Harefield Hospital, Royal Brompton & Harefield NHS Foundation Trust, Harefield, UK

^5^ Geisel School of Medicine, Dartmouth College, Hanover NH, USA

^6^ Department of Anesthesia and Intensive Care, Semmelweis University, Budapest, Hungary

^7^ Department of Chemical and Biological Engineering, University of British Columbia, Canada

^*^Corresponding author: Jane E. Hill, PhD; Tel: 1 (604) 861-4922; Fax: 1 (604) 861-6003; E-mail address: jane.hill@ubc.ca

^#^Joint first-authors

**CONTENT**

**METHODS……………….…..……………………………………………………………………………2**Sample collection…………………………………………………………………………………………2 Analytical conditions: GC×GC-TOFMS and SPME conditions…………………………………….……2 Pre-processing: Data reduction and quality assessment……..……………………...……………….……3 Processing: Model building and features selection…….……………………………………………………5

**RESULTS…………..…….…..……………………………………………………………………………6REFERENCES…………..…….…..………………………………………………………………………7**

**METHODS**

**Sample collection**

Samples from BALF and BBA were processed at the same time. A closed-suction system is routinely attached to the endotracheal tube of all lung transplant recipients in the intensive care units. The suction catheter was advanced to the maximal level, 20 mL of normal saline was flushed into the catheter, and then all saline/secretions were aspirated back through the catheter into suction traps. Samples were transferred on ice for processing; at collection time, butylated hydroxyl-toluene was added to every sample prior to filtering of debris and centrifugation at 1500 rpm, 10 min, 4 ˚C; samples were then frozen at -80 ˚C.

**Analytical conditions: GC×GC-TOFMS and SPME conditions**

The BALF samples were stored at -80 °C prior to sampling. They were unfrozen and 500 µL were transferred in a 20 mL headspace vial that were sealed with a PTFE/silicone cap (SIGMA-ALDRICH, St. Louis, MO). The samples were stored at -20 °C for a week until analysis. The volatile organic compounds present in the headspace were concentrated using a 2 cm Divinylbenzene/Carboxen/Polydimethylsiloxane (DVB/CAR/PDMS) solid-phase micro-extraction (SPME) fiber (film thickness: 50/30 μM) (SUPELCO, Bellenfonte, PA). The fiber was exposed to the headspace for 60 min at 37 °C with 250 rpm agitation.

Linear alkane standard (C_6_-C_17_) were also injected in order to determine the linear retention indices (LRIs) for identification purposes. ^1^

The GC×GC-TOFMS (Pegasus 4D, LECO Corporation, St. Joseph, MI) was equipped with an autosampler (MPS, GERSTEL, Linthicum Heights, MD). The column set consisted of an Rxi-624Sil MS (60 m × 250 μm × 1.4 μm (length × internal diameter × film thickness); RESTEK Corporation, Bellefonte, PA) first dimension and by a Stabilwax (Crossbond Carbowax polyethylene glycol; 1 m × 250 μm × 0.5 µm; RESTEK) second dimension. The primary oven ramped at 3.5 °C/min from 35 °C to 230 °C. The secondary oven, and modulator (2.0 s modulation period, 0.5 s alternating hot and cold pulses), were heated in step with the primary oven with +5 °C and +25 °C offsets, respectively. The helium carrier gas flow rate was 2 mL/min. A splitless injection was used, with a 180 s desorption time. The inlet and transfer line temperatures were set to 270 °C and 250 °C, respectively. Mass spectra were acquired over the range of 30 to 500 *m/z*, with an acquisition rate of 200 spectra/s. An acquisition delay of 170 s was applied (void time).

**Pre-processing: Data reduction and quality assessment**

*Data alignment and filtering*

The signal-to-noise ratio was set at 100 based on previous evaluation.^2^ NIST (2011) databases was employed for mass spectral identifications with a match factor threshold > 700. ^2,3^ Following this initial processing, the different chromatograms were aligned using the statistical compare feature of ChromaTOF (LECO). Every feature was aligned based the two retention times and the mass spectral information (peak-to-peak match of 700). A feature was rejected from the final table, if it was not identified in a least two samples. The resulting data matrix, containing 1336 features, was exported as *.CSV for future processing and statistical treatment. From the alignment output, different artifacts were removed: siloxanes, phthalates, compounds present in the blanks. The remaining features were checked one by one to insure that they result from real chromatographic signal. The cleaned data matrix, containing 386 features, will be used for all the following statistical treatment. Following the data reduction, the resulting matrix was normalized using probabilistic quotient normalization, mean centered and log transformed. ^4^

*Features identification verification*

In order to be identified, a feature has to: 1) reach a mass spectra match factor of 850 (85% similarity with the library); and 2) obtain a probability score above 6000 (60% probability) or retention indices with ± 5 units from the reference value (RESTEK modeler). The identification parameters and the name assignment are presented in Table SI-1.

**Table SI-1: 20 selected features from the SVM model and identification parameters**

|  | **Name** | **RI** | **Similarity** | **Reverse** | **Probability** |
| --- | --- | --- | --- | --- | --- |
| 1 | Alkylated hydrocarbon | - | - | - | - |
| 2 | 2-Propenoic acid, methyl ester | 642 | 960 | 960 | 9658 |
| 3 | Trichloromethane | 655 | 986 | 986 | 9205 |
| 4 | Ketone | 728 | - | - | - |
| 5 | Pentane, 2,3,3-trimethyl- | 760 | 918 | 918 | 6340 |
| 6 | Alkylated hydrocarbon | 767 | - | - | - |
| 7 | Heptane, 3-methyl- | 773 | 912 | 912 | 5372 |
| 8 | Alkylated hydrocarbon | 795 | - | - | - |
| 9 | Octane | 800* | 915 | 915 | 6138 |
| 10 | Alkylated hydrocarbon | 931 | - | - | - |
| 11 | Aldehyde | 943 | - | - | - |
| 12 | Alkylated hydrocarbon | 961 | - | - | - |
| 13 | Nonane, 3-methyl- | 973 | 904 | 904 | 5368 |
| 14 | Alkylated benzene | 1026 | - | - | - |
| 15 | Alkylated hydrocarbon | 1039 | - | - | - |
| 16 | Alkylated alcohol | 1141 | - | - | - |
| 17 | Nonanal | 1149 | 905 | 905 | 6581 |
| 18 | Undecane, 5-methyl- | 1156 | 884 | 884 | 2169 |
| 19 | Nitrogen cont. compounds | 1493 | - | - | - |
| 20 | Heptadecane | 1700* | 808 | 887 | 1067 |

*** Exact match of both retention times with the linear alkanes mix used for retention index determination**

*Technical replicates comparison*

For 10 patients, technical replicates were prepared. Pearson’s correlation coefficients ^5^ were all above 0.75, therefore technical replicates were combined for subsequent data processing. Missing peak values were replaced using small value imputation.^4^ The validity of this approach was assessed by evaluating the correlation significance between: peak area, missing count, and 1^st^ dimension retention time.

**Processing: Model building and features selection**

In order to identify the significant feature from the volatile organic compounds panel, we compared different algorithms: Random Forest (RF), Logistical Model Tree (LMT), and Support Vector Machine (SVM). For each algorithms, the model was build using leave-one-out cross validation (LOOCV) on the training set and the classification performance indicators were predicted on the test set. The classification accuracy and the area under the curve (AUROC) were used to compare the different models. SVM and RF provided us similar average accuracy and AUROC but the SVM was more stable regarding different train and test split of the data set (table below).

**
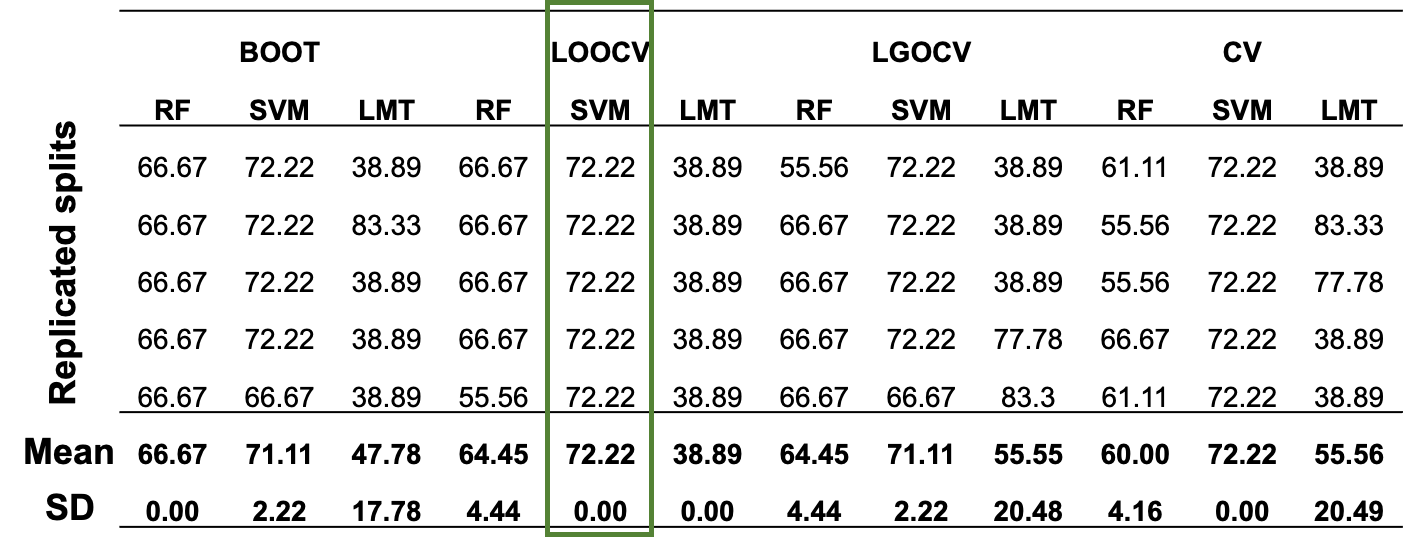
**

**Model comparison table:** Accuracy comparison for the different classification algorithms and cross-validation methods employed in this study using test set. The most stable and accurate method was the combination of SVM and LOOCV (green rectangle).

**R packages:** MetabolAnalyze, FactoMineR, ggfortify, randomForest, ggplot2, pca3d, mixOmics, car, grid, plyr, gridExtra, gplots, RColorBrewer, plot3D, ICC, psych, knitr. reshape2, Hmisc, corrgram, corrplot, caret, klaR, matrixStats, ROCR, LiblineaR, kernlab, e1071, lattice, beeswarm, ggpubr, pROC

**RESULTS**

**
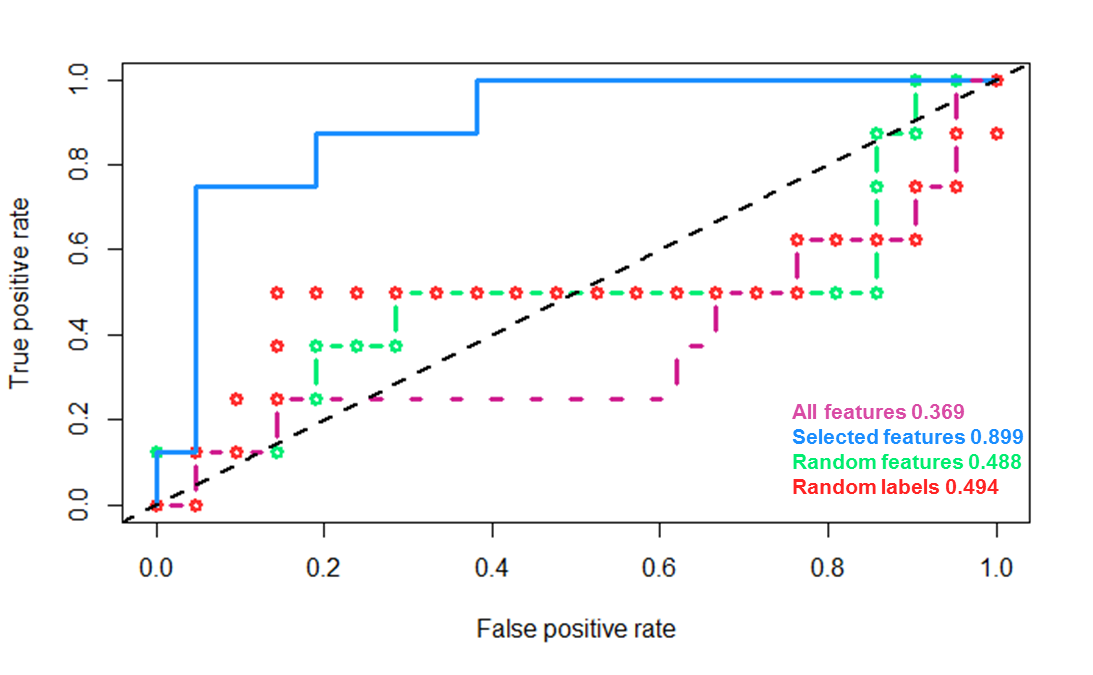
**

**Figure SI-1:** ROC and AUROC for the SVM models building. Several models were evaluated: 1. All features correspond to the full data matrix (386 features; 2. Selected features correspond to the final model used (20 features; 3. Random features correspond to a random selection of 20 features; 4. Random labels correspond to a random label assignment for the samples.

**Table SI-2:** Model performance indictor from the test set.

| **Model performance indicators** | **Values** |
| --- | --- |
| Accuracy | 0.828 |
| 95% Confidence interval | 0.683-0.961 |
| No information rate (NIR) | 0.621 |
| *p*-value (Accuracy > NIR) | 0.014 |
| Sensitivity | 0.636 |
| Specificity | 0.944 |
| Positive predictive value | 0.875 |
| Negative predictive value | 0.809 |

**Table SI-3:** Model performance indictor from the test set (sample collected between 6 and 72 hours post-transplant).

| **Model performance indicators** | **Values** |
| --- | --- |
| Accuracy | 0.714 |
| 95% Confidence interval | 0.528-0.873 |
| No information rate | 0.643 |
| *p*-value (Accuracy > NIR) | 0.401 |
| Sensitivity | 0.800 |
| Specificity | 0.667 |
| Positive predictive value | 0.571 |
| Negative predictive value | 0.857 |

**Table SI-4:** Selected features classification by chemical families

| **Name** | **Count of Name** |
| --- | --- |
| Fatty acid methyl ester (FAME | 1 |
| Alkylated alcohol | 1 |
| Nitrogen containing compounds | 1 |
| Halogenated compounds | 1 |
| Alkylated benzene | 1 |
| Ketone | 1 |
| Aldehyde | 2 |
| Linear hydrocarbon | 2 |
| Alkylated hydrocarbon | 10 |

**REFERENCES**

1. Zellner B d’Acampora, Bicchi C, Dugo P, Rubiolo P, Dugo G, Luigi Mondello. Linear retention indices in gas chromatographic analysis: a review. *Flavour Fragr J*. 2008;23(August):297-314. doi:10.1002/ffj

2. Stefanuto PH, Perrault KA, Dubois LM, et al. Advanced method optimization for volatile aroma profiling of beer using two-dimensional gas chromatography time-of-flight mass spectrometry. *J Chromatogr A*. 2017;1507:45-52. doi:10.1016/j.chroma.2017.05.064

3. Bean HD, Rees CA, Hill JE. Comparative analysis of the volatile metabolomes of *Pseudomonas aeruginosa* clinical isolates. *J Breath Res*. 2016;10(4):047102. doi:10.1088/1752-7155/10/4/047102

4. Di Guida R, Engel J, Allwood JW, et al. Non-targeted UHPLC-MS metabolomic data processing methods: a comparative investigation of normalisation, missing value imputation, transformation and scaling. *Metabolomics*. 2016;12(5):1-14. doi:10.1007/s11306-016-1030-9

5. Mukaka MM. Statistics corner: A guide to appropriate use of correlation coefficient in medical research. *Malawi Med J*. 2012;24(3):69-71. doi:10.1016/j.cmpb.2016.01.020
